# Expiratory central airway collapse during positive pressure ventilation: a case report

**DOI:** 10.1186/s12871-022-01591-y

**Published:** 2022-02-19

**Authors:** Guillaume Gaggini, Link-Mathieu Nkamicaniye, Sabrina Meyer, Philippe E. Dubois

**Affiliations:** Anaesthesiology department, University of Louvain, CHU UcL Namur, Avenue Dr Gaston Thérasse 1, B-5530 Yvoir, Belgium

**Keywords:** Expiratory central airway collapse, Airway obstruction, Desaturation, Case report

## Abstract

**Background:**

Physiologic narrowing of the central airway occurs during expiration. Conditions in which this narrowing becomes excessive are referred to as expiratory central airway collapse. Expiratory central airway collapse is usually managed by applying positive pressure to the airways, which acts as a pneumatic stent. The particularity of the case reported here included the patient’s left main bronchus being permeable during spontaneous breathing but collapsing during general anaesthesia, despite positive pressure ventilation and positive end-expiratory pressure.

**Case presentation:**

We present the case of a 55-year-old man admitted for the placement of a ureteral JJ stent. Rapid desaturation occurred a few minutes after the onset of anaesthesia. After excluding the most common causes of desaturation, fibreoptic bronchoscopy was performed through the tracheal tube and revealed complete collapse of the left main bronchus. The collapse persisted despite the application of positive end-expiratory pressure and several recruitment manoeuvres. After recovery of spontaneous ventilation, the collapse was lifted, and saturation increased back to normal levels. No evidence of extrinsic compression was found on chest X-rays or computed tomography scans.

**Conclusion:**

Cases of unknown expiratory central airway collapse reported in the literature were usually managed with positive pressure ventilation. This approach has been unsuccessful in the case described herein. Our hypothesis is that mechanical bending of the left main bronchus occurred due to loss of the patient’s natural position and thoracic muscle tone under general anaesthesia with neuromuscular blockade. When possible, spontaneous ventilation should be maintained in patients with known or suspected ECAC.

## Background

Physiologic narrowing of the central airway occurs during expiration. Conditions in which this narrowing becomes excessive are referred to as expiratory central airway collapse (ECAC). ECAC has a broad range of clinical presentations and is likely underdiagnosed [1]. It is usually managed with positive pressure ventilation, which acts as a pneumatic stent. Herein, we discuss the unusual clinical presentation of a patient with undiagnosed ECAC undergoing nonthoracic surgery under general anaesthesia.

## Case presentation

A 55-year-old man (95 kg, 170 cm, BMI 32,9 kg.m^− 2^) was admitted for emergency placement of a JJ stent. The patient had cerebral palsy, intellectual disability, deafness, and left hemiparesis due to prematurity and neonatal distress. He had acquired chronic obstructive pulmonary disease (COPD) with recurrent bronchial infections, pulmonary aspiration, and bronchospasms. He also had sleep apnoea syndrome but was not adherent to continuous positive airway pressure (CPAP) treatment.

Cardiac auscultation was normal, and pulmonary auscultation revealed clear, symmetrical vesicular murmurs, with some added rhonchi.

The anaesthesia plan consisted of general anaesthesia in the supine position with orotracheal intubation. After preoxygenation, the patient was given 7.5 μg of sufentanyl, 150 mg of propofol, and 35 mg of rocuronium intravenously. Orotracheal intubation occurred without incident, and auscultation showed bilateral and symmetrical pulmonary ventilation. SpO_2_ was 100%. The ventilator was set to pressure controlled ventilation – volume guaranteed mode with the following settings: respiratory rate 12.min^− 1^, maximum pressure 30 mmHg, tidal volume 500 mL, positive end-expiratory pressure (PEEP) 5 cm H_2_O. Anaesthesia was maintained with 1.5–2.0% sevoflurane.

A few minutes after induction, haemoglobin oxygen saturation rapidly dropped to 85%. Electrocardiogram was normal with a heart rate of 90 beats per minute, non-invasive blood pressure was 85/55 mmHg, FiO_2_ was 45%, end-tidal CO_2_ at 33 mmHg with a normal square curve, sevoflurane at a minimum alveolar concentration of 0.9. There was no significant change in peak inspiratory pressure. Based on these parameters, most causes of acute desaturation were quickly ruled out.

Manual ventilation was resumed with FiO_2_ 100%. On re-auscultation, the absence of ventilation of the left lung was observed. The first hypothesis was therefore selective intubation of the right main bronchus. The tube was withdrawn under direct laryngoscopic control until the tracheal cuff was positioned immediately under the vocal cords. The integrity and pressure inside the cuff were also checked.

**Fig. 1:**
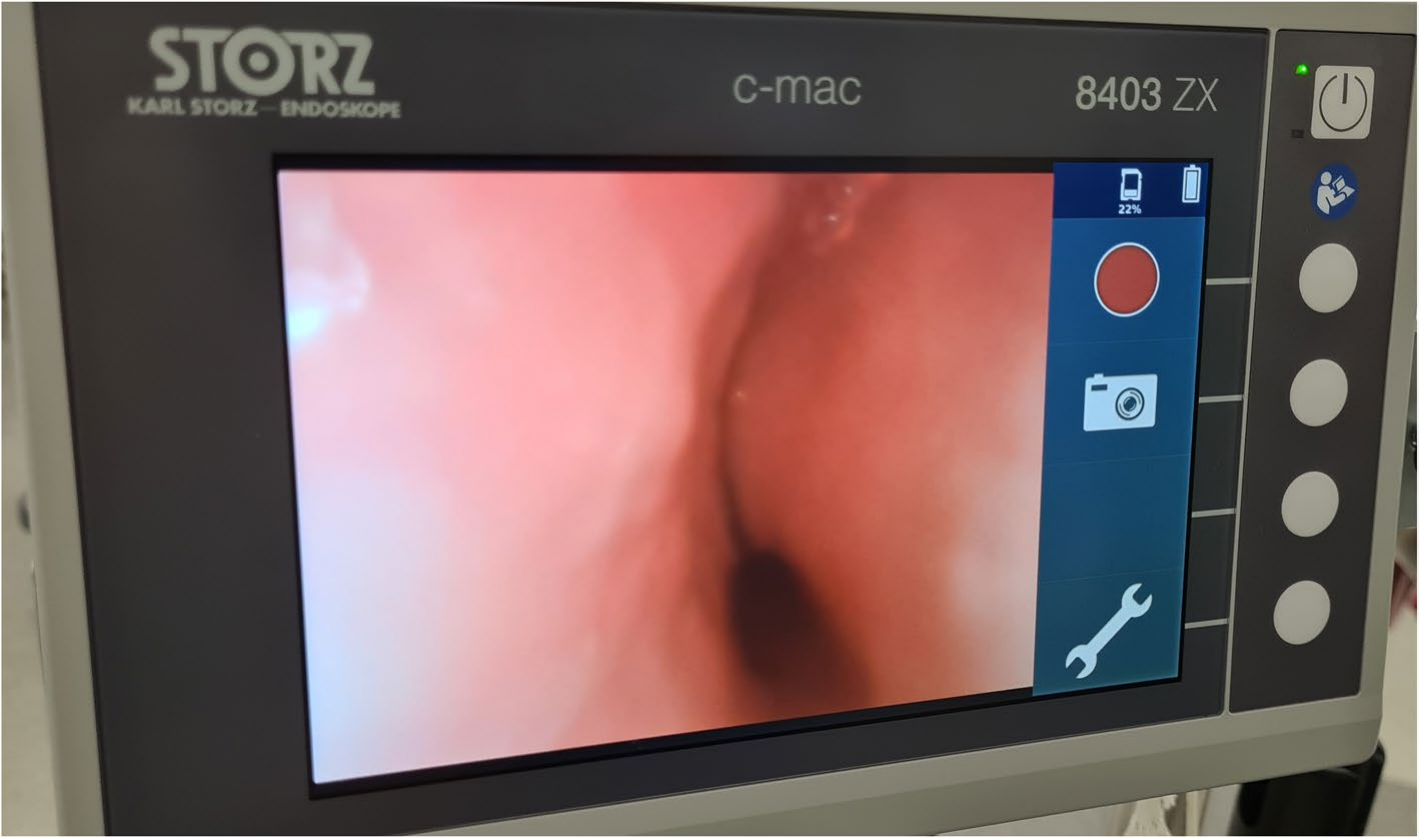
Collapse of the left main bronchus

Several recruitment manoeuvres (PEEP> 30 cm H_2_O for 40 s) were performed: SpO_2_ transiently increased to 94% before dropping again. Despite all of these interventions, left lung ventilation remained inaudible on auscultation, even during recruitment manoeuvres. Fibreoptic bronchoscopy through the tracheal tube revealed a complete collapse of the left main bronchus (Fig. 1). Interestingly, the airway downstream after the collapsed zone was permeable and free of secretion.

The surgical procedure lasted 30 min. Haemoglobin oxygen saturation was maintained above 90% by increasing FiO_2_ and performing several recruitment manoeuvres. After reversal of the neuromuscular block and sevoflurane washout, the patient recovered spontaneous ventilation. SpO_2_ quickly rose above 96%, and lung auscultation returned to normal with symmetrical vesicular murmur. At this point, the endotracheal tube was still in place. It was removed once standard criteria for extubation were met.

On re-reading the patient’s file after the procedure, we noticed that a similar episode of desaturation had already occurred during a previous anaesthesia but had not been further investigated. Of note, no evidence of extrinsic compression by an intrathoracic tumor, cyst, aortic aneurysm, vascular anomaly or goitre was found on recent chest X-rays and CT scans.

## Discussion and conclusions

Physiologic narrowing of the central airway occurs during expiration. Weakness in the structure of the trachea and/or the bronchi may cause this narrowing to become excessive. A clear cut-off between physiological and pathological narrowing has yet to be determined [2]. When extraluminal pressure exceeds intraluminal pressure, as during expiration or coughing, the affected areas may then collapse. These conditions are referred to as ECAC [1, 3, 4]. The causes of ECAC in adults are listed in Table 1.

**Table 1:** Causes of ECAC [1, 3]

Congenital	
• Tracheo-oesophageal fistula	
• Oesophageal atresia	
• Chromosomal defects	
• Tetralogy of Fallot	
• Genetic disorders	
Acquired	
• Compression by great vessels (Brachiocephalic, aorta, pulmonary artery)	
• Bronchopulmonary dysplasia	
• Pulmonary/mediastinal cysts	
• Intrathoracic tumors	
• Large/ectopic thymus	
• Goitres	
• Heart/lung transplant	
• Liver failure/transplant	
• Prolonged intubation	
• Tracheostomy	
• Emphysema	
• Chronic bronchitis/Chronic obstructive pulmonary disease	
• Relapsing polychondritis	
• Mucocutaneous leishmaniasis	

Non-invasive positive pressure ventilation is the cornerstone of treatment for ECAC. Positive pressure acts as a pneumatic stent, keeping airways open and improving respiratory flow. As a last resort, permanent mechanical stenting or surgical stabilization may be considered [1, 5].

If a patient with ECAC must undergo anaesthesia, spontaneous breathing should be maintained and CPAP applied. If not possible, PEEP should be applied during positive pressure ventilation. Airway collapse should be suspected in the following circumstances: hypoxia, increased insufflation pressures in volume control ventilation, and decreased tidal volume in pressure control ventilation. Recruitment manoeuvres should be attempted, and PEEP should be increased. Positional change should also be considered (reverse Trendelenburg or sitting position). If the obstruction persists, temporary mechanical stenting can be achieved by pushing the tracheal tube past the collapsed zone under fibreoptic bronchoscopy control. Jet ventilation can also be attempted. As a last resort, mechanical circulatory support should be considered [6].

A case report previously described an adult patient with cerebral palsy presenting with acute desaturation during the induction of anaesthesia [7]. Haemoglobin oxygen saturation rapidly improved with positive pressure ventilation. However, in spontaneous ventilation (negative airway pressure), desaturation occurred again. In this case, the cause was extrinsic compression of the trachea and left mainstem bronchus by the aorta.

Atkins et al. reported an 85-year-old patient with undiagnosed ECAC who was scheduled for Botox injections as a treatment for achalasia [8]. The patient began coughing during the procedure, and air flow stopped despite respiratory efforts, as assessed by paradoxical chest wall movements. Air flow was immediately restored by positive pressure ventilation. The main hypothesis was therefore that a collapse of the trachea occurred due to coughing.

In both cases described above, positive pressure ventilation improved gas flow and oxygen delivery. The particularity of the case reported here is the fact that the patient’s left main bronchus was permeable in spontaneous breathing but collapsed during general anaesthesia, including neuromuscular blockade. SpO2 increased slightly with recruitment manoeuvres (PEEP> 30 cm H2O for 40 s), suggesting that the collapse was at least partially lifted. However, even during these recruitment manoeuvres, auscultation showed a lack of effective ventilation of the left lung, indicating that even with high pressures, normal flow could not be restored. Our hypothesis is that mechanical bending of the left main bronchus occurred, leading to its complete obstruction. In spontaneous breathing, the patient would maintain a particular body position (slightly curved on its left side) and a thoracic muscle tone, keeping the left main bronchus permeable. Under anaesthesia, including neuromuscular blockade, this natural conformation was likely lost, and the bronchus bent until it collapsed. One of the difficulties in diagnosing ECAC resides precisely in the fact that the degree of collapse can vary depending on the respiratory pattern and the patient’s position [1]. If the patient was to present for longer procedures, a double lumen tube could be used, with the left endobronchial tube bypassing the lesion. The placement of a temporary or even permanent stent could also be considered.

The fact that we were not able to verify our hypothesis is a limitation to this case report. One way to do this would be to perform general anaesthesia while keeping the patient in spontaneous breathing. Fibreoptic bronchoscopy could then be performed to assess airway permeability while anaesthesia is deepened, and neuromuscular blockade induced.

In conclusion, applying positive pressure to the airways is the cornerstone of ECAC treatment. Interestingly, this approach has been unsuccessful in the case described herein. When possible, spontaneous ventilation should be maintained in patients with known or suspected ECAC.

## Data Availability

Not applicable.

## Abbreviations

COPD: Chronic obstructive pulmonary disease
CPAP: Continuous positive airway pressure
ECAC: Expiratory central airway collapse
PEEP: Positive end-expiratory pressure
